# Social Determinants of Health and Lifetime History of Parent-Reported Concussion in School-Aged Children and Adolescents in the United States

**DOI:** 10.1089/neur.2024.0083

**Published:** 2025-02-03

**Authors:** Grant L. Iverson, Julia E. Maietta, Altaf Saadi, Nathan E. Cook

**Affiliations:** ^1^ Department of Physical Medicine and Rehabilitation, Harvard Medical School, Boston, Massachusetts, USA.; ^2^ Department of Physical Medicine and Rehabilitation, Spaulding Rehabilitation Hospital and the Schoen Adams Research Institute at Spaulding Rehabilitation, Charlestown, Massachusetts, USA.; ^3^ Mass General for Children Sports Concussion Program, Waltham, Massachusetts, USA.; ^4^ Department of Physical Medicine and Rehabilitation, Spaulding Rehabilitation Hospital, Cambridge, Massachusetts, USA.; ^5^ Department of Neurology, Harvard Medical School, Boston, Massachusetts, USA.; ^6^ Department of Neurology, Massachusetts General Hospital, Boston, Massachusetts, USA.; ^7^ Department of Physical Medicine and Rehabilitation, Spaulding Rehabilitation Hospital, Charlestown, Massachusetts, USA.

**Keywords:** adversity, children, concussion, social determinants of health

## Abstract

Social determinants of health (SDoH) are environmental and socioeconomic factors that indirectly or directly influence health. This study examined whether SDoH that might relate to health literacy or access to health care are associated with lifetime history of parent-reported concussion in school-aged children and adolescents in the United States. We hypothesized that lower parental education, living in poverty, and speaking a language other than English as the primary language in the home would be associated with a lower lifetime history of concussion. Participants were parents or caregivers of 34,077 children and adolescents (ages 5–17) from the 2021 National Survey of Children’s Health. SDoH variables included primary language spoken at home, family income, parental level of education, and current health insurance. Univariable analyses assessed the individual association of each SDoH variable with lifetime history of concussion. A multivariable logistic regression was used to assess the combined association of SDoH variables and other demographic predictors with lifetime concussion history. In the univariable models, male gender, older age, sports participation, and having current health care coverage were associated with a higher lifetime history of concussion. Hispanic/Latino ethnicity, primary language spoken at home other than English, lower level of parental education, living in poverty, and Black or Asian race were associated with lower lifetime history of concussion. In a multivariable model, significant independent predictors of lower lifetime concussion history were lower level of parental education, not speaking English as the primary language at home, and identifying as Black or Asian. It is possible that lower parental education, living in poverty, and speaking a language other than English as the primary language spoken are factors relating to lower concussion-related health literacy. Lower health literacy might contribute to families being less likely to (i) recognize the symptoms of concussion and (ii) seek medical evaluation for the injury.

## Introduction 

Social determinants of health (SDoH) and their contributions to child and adolescent health outcomes and health inequity have been well established for decades,^1,2^ but they remain understudied in the literature relating to pediatric concussion.^3^ SDoH include a diverse range of conditions, present in the environments in which we are born, raised, and live, that indirectly or directly influence health.^4,5^ Examples include parental education, family functioning (connectedness, norms and attitudes toward health behaviors, parental health behaviors, parental monitoring of youth behavior), income or socioeconomic status, access to health care, neighborhood deprivation and safety (air and water quality, safe places to exercise, safe for pedestrians), and racism.^1,5–7^ Adverse SDoH and social risk factors disproportionately impact minoritized racial and ethnic groups, with structural racism as a root cause of these disproportionate social risks.^8^

There is accumulating evidence for racial, ethnic, and socioeconomic disparities associated with concussion-related health literacy, such as concussion knowledge, awareness of concussion symptoms, and familiarity with concussion laws relating to medical clearance for return to sports^9–13^ as well as access to concussion health care.^13–16^ Health literacy refers to “the degree to which individuals have the ability to find, understand, and use information and services to inform health-related decisions and actions for themselves and others.”^17,18^ Health care access and health literacy are associated with insurance status, higher income and socioeconomic status,^19,20^ English language proficiency,^21,22^ and higher educational attainment.^23,24^

The purpose of this study is to determine whether social risks, influenced by SDoH (hereinafter “SDoH variables”), that might relate to health care access and health literacy are associated with lifetime history of parent-reported concussion in school-aged children in the United States, including differences by gender. Using a national survey conducted by the United States Census Bureau, we examined the associations between (i) parental level of education, (ii) living in poverty, (iii) primary language spoken at home, and (iv) lifetime history of concussion in school-aged children and adolescents. We hypothesized that lower parental education, living in poverty, and speaking a language other than English as the primary language in the home would be associated with a *lower* lifetime history concussion. The possible mechanism for this lower parent-reported lifetime history, if present, might relate to health literacy and access to health care to receive a concussion diagnosis (i.e., being less likely to realize that a head injury might be a concussion and thus not seeking health care for the injury).

## Method

### Survey methodology and participants

Participants were selected from the 2021 National Survey of Children’s Health (NSCH). These national survey data are collected and disseminated by the U.S. Department of Health and Human Services’ Child and Adolescent Health Measurement Initiative.^25^ Households were randomly selected and contacted by mail to participate in the survey. Parents or other adult caregivers who were knowledgeable about a child living in the home responded to survey questions related to the child’s demographics, physical and mental health status, family health and activities, and other sociodemographic characteristics.^25^ Thus, the survey respondents were parents and caregivers, but the data/results presented refer to their children’s health. When multiple children were present in a household, one child was randomly selected as the subject of the survey. We selected data for 34,077 children and adolescents (ages 5–17) who did not have missing data on the primary concussion question.

### Variables of interest

Several variables of interest were identified. The precise variable names from the NSCH dataset and the exact question wording in the NSCH survey for each variable of interest are presented in Table 1.

**Table 1.** National Survey of Children’s Health Variable Names

Variable description	Variable name in 2021 NSCH data	Question in the survey
Concussion history	CONCUSSION	Do you think this child has ever had a concussion or brain injury? A concussion or brain injury is when a blow or jolt to the head causes problems such as headaches, dizziness, being dazed or confused, difficulty remembering or concentrating, vomiting, blurred vision, changes in mood or behavior, or being knocked out.
Concussion—seeking care	SEEKCARE	Did you seek medical care from a doctor or other health care provider?
Concussion—clinician confirmed injury	CONFIRMINJURY	Did a doctor or other health care provider tell you that this child had a concussion or brain injury?
Sports participation	K7Q30	During the past 12 months, did this child participate in: A sports team or did they take sports lessons after school or on weekends?
Race	SC_RACE_R	What is this child’s race?
Ethnicity	SC_HISPANIC_R	Is this child of Hispanic, Latino, or Spanish origin?
Age	SC_AGE_YEARS	How old is this child? [in years]
Sex	SC_SEX	What is this child’s sex?
Health insurance coverage	CURRCOV	Is this child CURRENTLY covered by ANY kind of health insurance or health coverage plan?
Primary language	HHLanguage_21	What is the primary language spoken in the household?
Parental level of education	HIGRADE_TVIS	This variable is derived from two questions, each asking about one parent/guardian’s level of education. This variable reflects the highest level of education from both parents/guardians.
Family income	WrkngPoorR_21	Does this child live in a “working poor” household: that is, a household with income less than 100% of the federal poverty level and at least one caregiver employed full- or part-time?

NSCH, National Survey of Children’s Health.

#### Concussion History

The NSCH survey question assessing concussion history is: “Do you think this child has EVER had a concussion or brain injury? A concussion or brain injury is when a blow or jolt to the head causes problems such as headaches, dizziness, being dazed or confused, difficulty remembering or concentrating, vomiting, blurred vision, changes in mood or behavior, or being knocked out.”

#### Demographics

The NSCH dataset includes the following categories for children’s race: White, Black/African American, Native Hawaiian or Other Pacific Islander, American Indian or Alaska Native, Asian, or two or more races. Ethnicity was reported as Hispanic origin or non-Hispanic origin. Age was provided via numeric entry and was dichotomized for some analyses to represent child (ages 5–≤12) and adolescent (ages ≥13–17) age ranges. Sex was reported as either male or female (relabeled for this study as gender with the categories “boys” and “girls”).

#### Sports Participation

Parents or caregivers were also asked whether their child participated in sports within the past 12 months (i.e., “During the past 12 months, did this child participate in a sports team or did they take sports lessons after school or on weekends?”). There were 4,434 (13.0%) missing cases for this variable. The demographic characteristics (age, gender, and racial or ethnic group) were not significantly different from nonmissing cases. The demographic characteristics of those with missing data on this variable were as follows: boys = 12.9%, girls = 13.2%, Hispanic/Latino = 13.1%, White = 12.9%, Black/African American = 12.8%, Native Hawaiian or Other Pacific Islander = 9.2%, American Indian or Alaska Native = 10.5%, Asian = 14.8%, or two or more races = 13.6%.

#### Health Insurance

Parents and caregivers were asked if their child was currently covered by health insurance at the time of the survey (i.e., “Is this child currently covered by any kind of health insurance or health coverage plan?” with response options being yes and no). There were missing data for 147 cases (0.4%) on this variable. There were no questions that asked about multiyear or lifetime access to health care.

#### Language Spoken at Home

Response options for the primary language spoken at home included English, Spanish, and other languages. The question relating to primary language was as follows: “What is the primary language spoken in the household?” There were 185 (0.5%) missing cases for this variable. This variable was used as a proxy for English language proficiency (such that English being the primary language spoken in the home was presumed to indicate higher levels of English language proficiency at the family level in comparison to families indicating Spanish or another language as the primary language spoken in the home).

#### Parental Level of Education

The highest level of parental education variable combines responses that relate to both parents/caregivers to determine the highest level of education between them. Response options included: 8th grade or less, 9th–12th grade (no diploma), high school graduate or GED (General Educational Development), vocational/trade/business school program, some college credit (no degree), associate’s degree, bachelor’s degree, master’s degree, and doctorate or professional degree. These categories were collapsed by the NSCH developers into a four-level variable used for this study: (i) less than high school education, (ii) high school or GED, (iii) some college or technical school (no college degree), and (iv) college degree or higher.

#### Living in Poverty

Living in poverty was classified if parent or caregiver(s) indicated that the household income was less than 100% of the federal poverty level with at least one caregiver employed full- or part-time. This variable, considered “working poor,” was created by the NSCH developers, and it combines income questions with questions based on both caregivers’ working status. There were 1,171 (3.4%) missing cases for this variable.

### Statistical analyses

Logistic regressions were conducted with parent-reported lifetime history of concussion in their child as the dichotomous dependent variable. The analyses were conducted for the total sample and separately for girls and boys. The analyses were conducted separately by gender because of gender differences in concussion prevalence documented in past studies^26,27^ and because it is desired to be able to draw conclusions about associations within each gender. Ten demographic and SDoH variables were the predictor variables (see Tables 1 and 3). An odds ratio (OR) above 1.00 with a 95% confidence interval (CI) not including 1.00 indicated the predictor was significantly associated with greater odds of having a lifetime history of concussion, whereas an OR below 1.00 with a 95% CI not including 1.00 indicated the predictor was associated with reduced odds of having a lifetime history of concussion. A multivariable logistic regression was conducted, including all predictors in the same model to examine the independence and magnitude of their associations. The ORs can be interpreted in the same manner, but they reflect the increase or decrease in odds of having a lifetime history of concussion after adjusting for all other variables in the model. Of note, we were not attempting to build causal models with these analyses, nor were we attempting to understand the interrelationships among these variables. Instead, we were examining which variables are associated with lifetime history of concussion in the multivariable model.

## Results

**FIG. 1.**
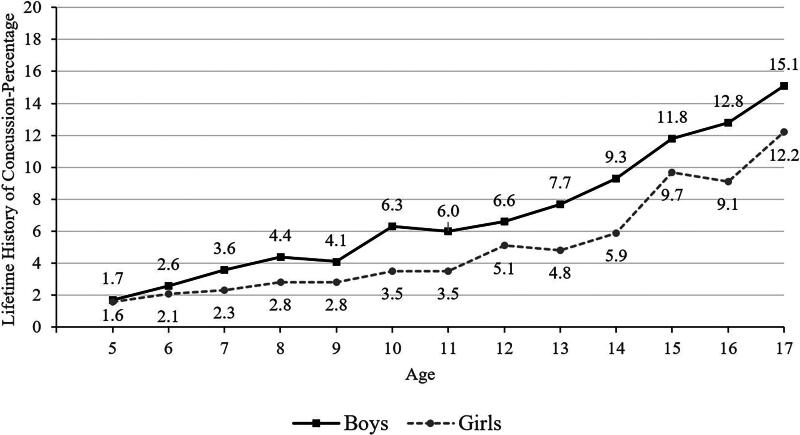
Lifetime history of concussion by age and gender.

**FIG. 2.**
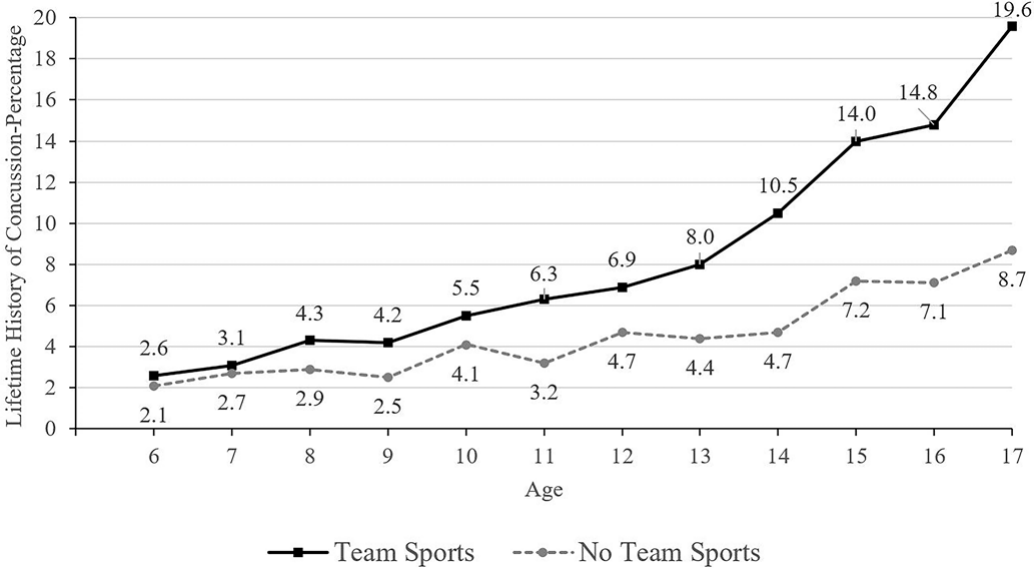
Lifetime history of concussion by age and participation in team sports in the past year.

**FIG. 3.**
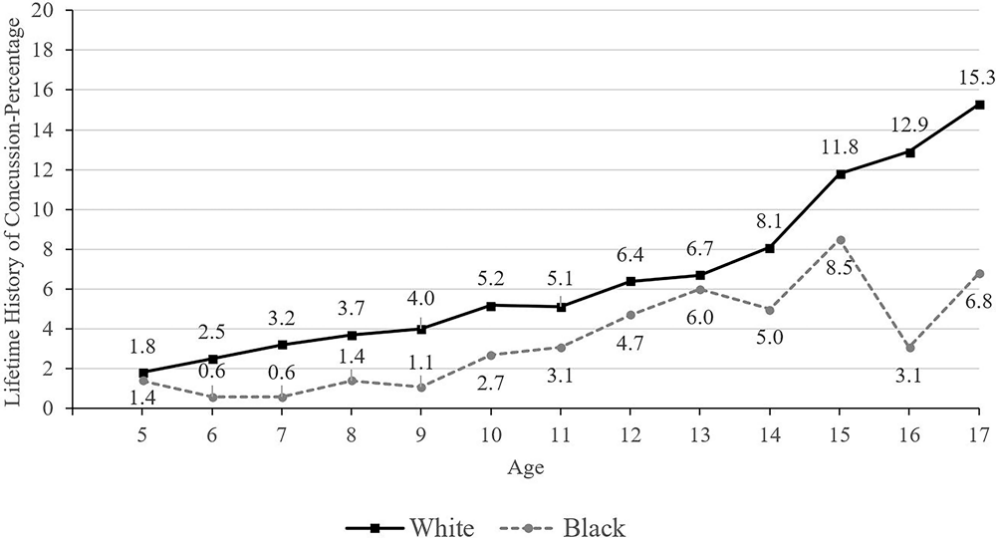
Lifetime history of concussion by age and race.

**FIG. 4.**
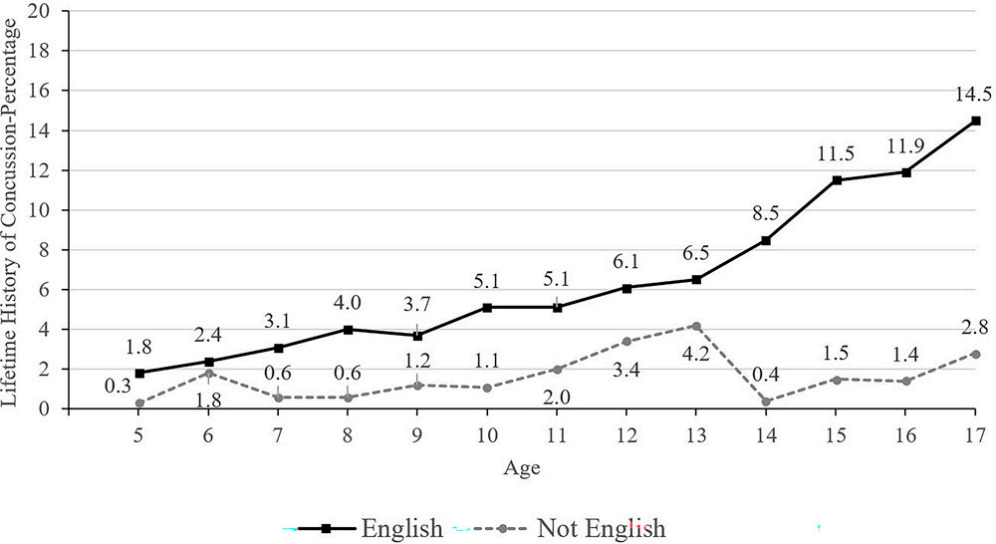
Lifetime history of concussion by age and primary language spoken at home.

**FIG. 5.**
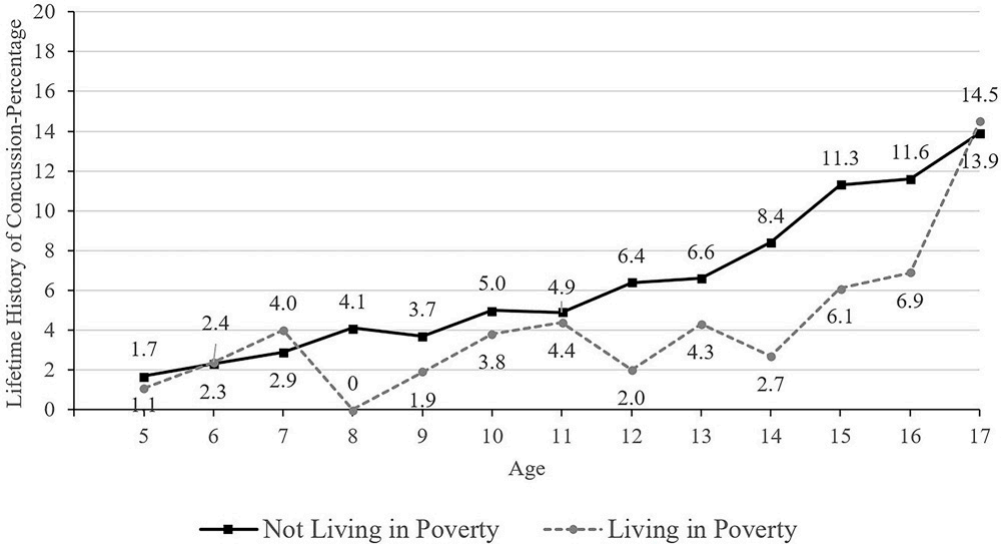
Lifetime history of concussion by age and family living in poverty.

**FIG. 6.**
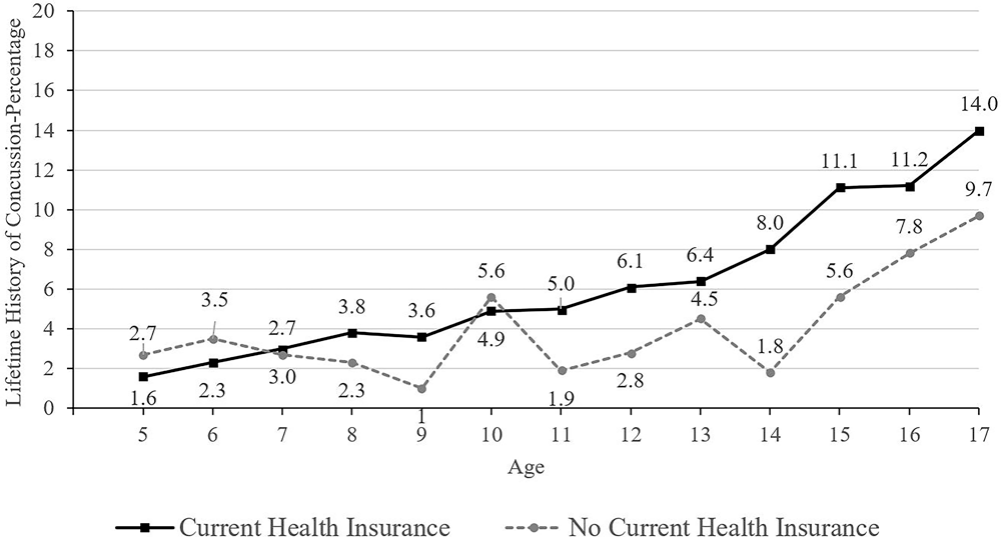
Lifetime history of concussion by age and current health insurance coverage.

Parents and caregivers reported data for their children who were, on average, 11.0 years of age (*SD* = 4.0, range = 5–17). Approximately half the youth (47.9%) were girls and half (52.1%) were boys. Children’s racial identities were: 76.3% White, 7.4% Black, 1.1% American Indian/Alaska Native, 5.9% Asian, 0.8% Native Hawaiian/Other Pacific Islander, and 8.4% reporting two or more races. Hispanic or Latino ethnicity was reported for 13.7% of the youth. Lifetime histories of concussion for the total sample, among different subsamples (e.g., adolescent boys), and stratified by SDoH variables are presented in Table 2. Estimates of parent-reported lifetime concussion history across different ages and across the variables of interest (sex, sports participation, race, primary language, living in poverty, and health insurance coverage status) are presented in Figures 1–6.

**Table 2.** Lifetime History of Concussion by Demographic Variables

Variable	Total sample			Boys			Girls		
	n	f	%	n	f	%	n	f	%
Total sample	34,077	2,109	6.2%	17,767	1,267	7.1%	16,310	842	5.2%
Children (ages 5–12)	20,229	710	3.5%	10,551	436	4.1%	9,678	274	2.8%
Adolescents (ages 13–17)	13,848	1,399	10.1%	7,216	831	11.5%	6,632	568	8.6%
Sports participation									
Yes	15,796	1,369	8.7%	8,609	862	10.0%	7,187	507	7.1%
No	13,847	659	4.8%	6,871	362	5.3%	6,976	297	4.3%
Race									
White	26,014	1,769	6.8%	13,620	1,066	7.8%	12,394	703	5.7%
Black/African American	2,528	92	3.6%	1,314	58	4.4%	1,214	34	2.8%
American Indian/Alaska Native	380	22	5.8%	201	17	8.5%	179	5	2.8%
Asian	2,023	42	2.1%	1,032	18	1.7%	991	24	2.4%
Native Hawaiian/Other Pacific Islander	261	8	3.1%	137	4	2.9%	124	4	3.2%
Two or more races	2,871	176	6.1%	1,463	104	7.1%	1,408	72	5.1%
Hispanic/Latino origin									
Yes	4,666	212	4.5%	2,443	122	5.0%	2,223	90	4.0%
No	29,411	1,897	6.4%	15,324	1,145	7.5%	14,087	752	5.3%
Primary language									
English	31,198	2,056	6.6%	16,238	1,235	7.6%	14,960	821	5.5%
Other	2,694	43	1.6%	1,427	23	1.6%	1,267	20	1.6%
Parental education									
Less than high school	988	25	2.5%	533	16	3.0%	455	9	2.0%
High school	4,764	220	4.6%	2,547	138	5.4%	2,217	82	3.7%
Some college/associate’s degree	7,575	472	6.2%	3,912	288	7.4%	3,663	184	5.0%
College degree or higher	20,750	1,392	6.7%	10,775	825	7.7%	9,975	567	5.7%
Living in poverty									
Yes	3,141	131	4.2%	1,639	81	4.9%	1,502	50	3.3%
No	29,765	1,927	6.5%	15,494	1,148	7.4%	14,271	779	5.5%
Current health insurance coverage									
Insured	32,490	2,044	6.3%	16,897	1,222	7.2%	15,593	822	5.3%
Uninsured	1,440	62	4.3%	790	43	5.4%	650	19	2.9%

*n* = sample size. *f* = number with parent-reported lifetime history of concussion. The percentage (%) columns represent the proportion of that subgroups with a parent-reported lifetime history of concussion. The survey question was: “Do you think this child has EVER had a concussion or brain injury?” and the parents and caregivers were provided a definition of the injury (see the Methods section).

The percentages of youth with a history of concussion are presented in Table 2. As seen in Table 3, a statistical test for proportional differences is presented for many of those individual characteristics (i.e., the unadjusted logistic regression analyses with ORs and 95% CIs). Of the 34,077 children and adolescents, 6.2% had a parent or caregiver-reported lifetime history of concussion (*n* = 2,109; Table 2). See Figure 1 for lifetime history of concussion stratified by gender and by age. Adolescents were more likely than children to have a history of concussion (10.1% versus 3.5% of children; Tables 2 and 3, and Fig. 1).

**Table 3.** Prediction of Lifetime Concussion History in Unadjusted and Adjusted Analyses

	Multivariable analyses							Univariable unadjusted analyses			
						95% CI				95% CI	
Total sample	B	S.E.	Wald	p	OR	Lower	Upper	p	OR	Lower	Upper
Gender	0.328	0.048	46.220	**0.000**	**1.388**	1.263	1.526	0.000	**1.411**	1.289	1.543
Binary age groups: children versus adolescents	1.060	0.051	439.443	**0.000**	**2.886**	2.614	3.186	0.000	**3.089**	2.815	3.391
Identifies as Hispanic or Latino	−0.107	0.082	1.682	0.195	0.899	0.765	1.056	0.000	**0.690**	0.597	0.798
English not primary language spoken at home	−1.033	0.171	36.318	**0.000**	**0.356**	0.254	0.498	0.000	**0.230**	0.170	0.312
Identifies as Black	−0.613	0.118	27.164	**0.000**	**0.542**	0.430	0.682	0.000	**0.553**	0.447	0.684
Identifies as Asian	−1.020	0.169	36.365	**0.000**	**0.361**	0.259	0.502	0.000	**0.308**	0.226	0.419
Sports team or sports lessons—past 12 months	0.548	0.052	113.313	**0.000**	**1.730**	1.564	1.914	0.000	**1.899**	1.725	2.090
Highest level of education among reported adults	−0.084	0.034	6.008	**0.014**	**0.919**	0.859	0.983	0.000	**0.816**	0.770	0.865
Living in poverty	−0.174	0.099	3.105	0.078	0.841	0.693	1.020	0.000	**0.629**	0.525	0.753
Current health insurance coverage	0.272	0.150	3.295	0.070	1.313	0.979	1.761	0.002	**1.492**	1.152	1.932
Constant	−3.710	0.172	463.949	0.000	0.024	—	—	—	—	—	—

	Multivariable analyses							Univariable unadjusted analyses			
						95% CI				95% CI	
**Girls**	B	S.E.	Wald	p	OR	Lower	Upper	p	OR	Lower	Upper
Binary age groups: children versus adolescents	1.121	0.080	194.123	**0.000**	**3.067**	2.620	3.591	0.000	**3.215**	2.773	3.727
Identifies as Hispanic or Latino	0.020	0.125	0.027	0.870	1.021	0.799	1.304	0.011	**0.748**	0.598	0.935
English not primary language spoken at home	−0.915	0.250	13.449	**0.000**	**0.400**	0.245	0.653	0.000	**0.276**	0.177	0.432
Identifies as Black	−0.630	0.189	11.087	**0.001**	**0.533**	0.368	0.772	0.000	**0.510**	0.360	0.722
Identifies as Asian	−0.637	0.215	8.736	**0.003**	**0.529**	0.347	0.807	0.000	**0.440**	0.292	0.664
Sports team or sports lessons—past 12 months	0.430	0.078	30.067	**0.000**	**1.537**	1.318	1.793	0.000	**1.707**	1.473	1.977
Highest level of education among reported adults	−0.134	0.055	5.945	**0.015**	**0.875**	0.786	0.974	0.000	**0.793**	0.722	0.871
Living in poverty	−0.284	0.160	3.157	0.076	0.753	0.550	1.030	0.000	**0.596**	0.446	0.798
Current health insurance coverage	0.350	0.247	2.018	0.155	1.420	0.875	2.302	0.009	**1.848**	1.165	2.933
Constant	−3.700	0.274	181.806	.000	.025	—	—	—	—	—	—

	Multivariable analyses							Univariable unadjusted analyses			
						95% CI				95% CI	
**Boys**	B	S.E.	Wald	p	OR	Lower	Upper	p	OR	Lower	Upper
Binary age groups: children versus adolescents	1.022	0.065	246.503	**0.000**	**2.780**	2.447	3.159	0.000	**3.019**	2.678	3.405
Identifies as Hispanic or Latino	−0.199	0.109	3.312	0.069	0.820	0.662	1.015	0.000	**0.651**	0.537	0.788
English not primary language spoken at home	−1.119	0.236	22.528	**0.000**	**0.327**	0.206	0.519	0.000	**0.199**	0.131	0.302
Identifies as Black	−0.609	0.150	16.402	**0.000**	**0.544**	0.405	0.730	0.000	**0.582**	0.445	0.762
Identifies as Asian	−1.460	0.275	28.150	**0.000**	**0.232**	0.135	0.398	0.000	**0.220**	0.138	0.352
Sports team or sports lessons—past 12 months	.637	0.069	86.222	**0.000**	**1.890**	1.652	2.162	0.000	**2.001**	1.762	2.272
Highest level of education among reported adults	−0.050	0.044	1.257	0.262	0.951	0.872	1.038	0.000	**0.828**	0.769	0.892
Living in poverty	−0.100	0.126	0.633	0.426	0.905	0.708	1.157	0.000	**0.650**	0.516	0.819
Current health insurance coverage	0.225	0.189	1.414	0.234	1.252	0.864	1.815	0.057	1.354	0.990	1.852
Constant	−3.410	0.217	245.799	0.000	0.033	—	—	—	—	—	—

Values in **bold **indicate statistically significant results (*p* < .05). Total sample (*N* = 34,077): χ^2^(10) = 879.852, *p* < 0.001, Cox & Snell *R*^2^ = 0.030, Nagelkerke *R*^2^ = 0.077. For girls (*n* = 16,310): χ^2^(9) = 328.817, *p* < 0.001, Cox & Snell *R*^2^ = 0.024, Nagelkerke *R*^2^ = 0.067. For boys (*n* = 17,767): χ^2^(9)=515.139, *p* < 0.001, Cox & Snell *R*^2^ = 0.034, Nagelkerke *R*^2^ = 0.080.

S.E., standard error; CI, confidence interval; OR, odds ratio.

Those children and adolescents identified as Black/African American or Asian race were less likely to have a concussion history (3.6% for Black participants and 2.1% for Asian participants, compared to 6.8% for White participants; Tables 2 and 3). Primary English speakers (6.6%) were much more likely to have a concussion history than those who spoke a language other than English in the home (1.6%; Tables 2 and 3). Those having health insurance coverage were more likely to have a prior concussion (6.3% versus 4.3% without coverage; Tables 2 and 3, and Fig. 6). Parents/caregivers with less than a high school education reported that their children and adolescents (2.5%) had a lower lifetime history of concussion compared to children and adolescents of parents with a college degree or higher (6.7%; Table 2). Those living in poverty (4.2%) were less likely to have a concussion history compared to those who were not working poor households (6.5%; Tables 2 and 3).

The results of the unadjusted univariable and adjusted multivariable models are presented in Table 3 for the total sample and for boys and girls separately. In univariable logistic regressions, boys (OR = 1.41; 95% CI = 1.29–1.54), adolescents (i.e., 13–17 [OR = 3.09; 95% CI = 2.82–3.39]), youth who played a sport in the past 12 months (OR = 1.90; 95% CI = 1.73–2.09), and youth with current health care insurance (OR = 1.49; 95% CI = 1.15–1.93) had significantly higher lifetime prevalence of concussion. Hispanic/Latino youth (OR = 0.69; 95% CI = 0.60–0.80), youth from a home where English is not the primary language spoken (OR = 0.23; 95% CI = 0.17–0.31), youth from families with lower levels of parental education (OR = 0.82; 95% CI = 0.77–0.87), youth living in poverty (OR = 0.63; 95% CI = 0.53–0.75), and youth identified as Black (OR = 0.55; 95% CI = 0.45–0.68) or Asian (OR = 0.31; 95% CI = 0.23–0.42) had significantly lower lifetime prevalence of concussion. In the multivariable model for the total sample, after accounting for the combined effects of all predictors (χ^2^[10]=879.85, *p* < .001, Nagelkerke *R*^2^ = 0.077), significant independent predictors of lower lifetime concussion prevalence were lower level of parental education (OR = 0.92; 95% CI = 0.86–0.98), speaking a language other than English as the primary language in the home (OR = 0.36; 95% CI = 0.25–0.50), and identifying as Black (OR = 0.54; 95% CI = 0.43–0.68) or Asian (OR = 0.36; 95% CI = 0.23–0.50). Male gender (OR = 1.39; 95% CI = 1.26–1.53), older age (OR = 2.89; 95% CI = 2.61–3.19), and sports participation within the past year (OR = 1.73; 95% CI = 1.56–1.91) were associated with higher lifetime concussion prevalence. The results were very similar when analyses were conducted separately for boys and for girls (see Table 3).

## Discussion

Several demographic and SDoH variables were associated with parent-reported lifetime history of concussion in this nationwide study of children and adolescents in the United States. We hypothesized that lower parental education, living in poverty, and speaking a language other than English as the primary language in the home would be associated with a *lower* lifetime history concussion—possibly because of lower concussion health literacy and less access to health care. In multivariable analyses, these variables were associated with a lower parent-reported lifetime concussion prevalence, although living in poverty was not a significant independent predictor. In addition, younger age, female gender, Black race, Asian race, and Hispanic ethnicity were associated with a lower parent-reported lifetime concussion prevalence.

In school-aged children, ages 5–17, 6.2% of their parents reported that they have a lifetime history of concussion, including 3.5% for those between 5 and 12 versus 10.1% for those between 13 and 17 years old. There was a strong association with age, in that fewer than 2% of 5-year-olds and 12–15% of 17-year-olds had a lifetime history of concussion (see Fig. 1). Fewer children identified as Black (3.6%), Asian (2.1%), and Hispanic (4.5%) had lifetime histories of concussion compared to those identified as White (6.8%). Youth who participated in sports during the past year (8.7%) have a greater lifetime history of concussion than those who did not participate in sports (4.8%). Adjusting for all of these variables in multivariable analyses, as hypothesized, lower parental education and speaking a language other than English as the primary language spoken at home were independently associated with lower lifetime history of concussion.

It is possible that some subgroups of children and adolescents in the United States have a lower lifetime prevalence of concussion because they have less access to health care. Disparities in specialty concussion and neurological care access have been identified.^14,28,29^ This may result in a lower likelihood of being medically diagnosed with concussion. At the same time, some subgroups of children and adolescents may have lower participation rates in sports that carry an increased risk for concussion.^30,31^ This could be influenced by social disadvantage, for example, certain sports might involve participation fees and may require that families purchase equipment that might be expensive (e.g., protective equipment, specialized footwear, or other gear). Disparities in access to medical coverage by athletic trainers have been reported in schools with greater proportions of students qualifying for free and reduced lunch.^32^ Less access to athletic trainers might result in student athletes being less likely to be identified as having sustained a concussion.

Speaking a language other than English as the primary language at home was associated with a lower lifetime history of concussion in youth. Adults with limited English language proficiency have worse access to health care,^33^ and they are underrepresented in most outpatient specialty practices—especially medical subspecialties and surgical specialties.^34^ Adults with limited English proficiency have more social needs in general, including material need, employment, health insurance, health literacy, medical care, poor housing quality, and food insecurity.^21^ When parents have limited English language proficiency, this is associated with worse health care access and quality for children with special health care needs.^22^ Thus, these families might have less access to health care for concussion.

Families with lower parental education and those speaking a language other than English also have greater likelihood of lower health literacy and, specifically, lower concussion health literacy.^35^ Lower health literacy might contribute to families being less likely to recognize the symptoms of concussion and to seek medical evaluation for the injury. For example, prior studies have identified racial disparities in concussion knowledge among high school student athletes^10^ and parents of high school student athletes.^11^ Therefore, parents with lower health literacy might report a lower lifetime history of concussion in their children. We did not examine health literacy in general, or concussion-related health literacy, in this study. No questions relating to these topics were included in the survey. Indirectly, however, we might infer that lower parental education, living in poverty, and speaking a language other than English as the primary language spoken in the household are factors that could be associated with lower concussion-related health literacy. Future studies could specifically assess health literacy and concussion-related health literacy among diverse populations.

### Limitations

This study has important limitations. All data were self-reported by parents and caregivers. The accuracy of the information could not be independently verified, and it could be subject to different sources of bias and recall bias. Estimates of annual incidence and lifetime prevalence of concussion vary depending upon the source of the information. For example, in a national survey of more than 13,000 adolescents in the United States, 13% self-reported experiencing a concussion in the past year.^36^ In a sample of more than 32,000 adolescents undergoing preseason sports participation testing, 17% reported a lifetime history of one or more concussions.^37^ Similar to prior studies, lifetime history of concussion was documented through a single question, and the reliability (i.e., reproducibility) and accuracy of that question and the resulting data are unknown. There was no information relating to the severity of the injury to the brain, the timing of the injury (age of injury or time between the injury and the caregiver completing the questionnaire), or whether the child or adolescent experienced a swift versus prolonged clinical recovery. The health insurance variable related to having *current* health insurance over the past year, and there was no information relating to access to health insurance over the course of the child’s lifetime. Similarly, family income information was based on the past year only. This prevented more refined analyses relating to longitudinal trajectories of socioeconomic status and associations with concussion. Additionally, language spoken at home was used as a proxy variable for English language proficiency due to limited variables available in the current dataset. Certainly, other measures of language proficiency would better characterize the extent of English language proficiency.^38^ The associations identified in this cross-sectional study should not be considered causal. In the total sample, the multivariable model with 10 variables accounted for only 8% of the variance in lifetime history of concussion, indicating that there is a tremendous amount of variance that remains unexplained (see the table note under Table 3).

## Conclusions

When considered independently, several SDoH and associated risk factors were related to a lower lifetime prevalence of parent-reported concussions in youth in the United States, as reported by their parents and caregivers. These included, but were not limited to, Asian race, Black race, speaking a language other than English as the primary language at home, and parental education less than high school. Living in poverty and not having current health insurance were related to a lower lifetime prevalence of concussion—and there might be ages during which this is more pronounced (e.g., ages 12–14; see Figs. 5 and 6). Those two variables, however, were not significantly associated with lifetime prevalence of concussion after adjusting for a broad range of other variables in a multivariable model. The results of this study have possible implications for child and adolescent health care equity. There might be subgroups of youth who have a lower lifetime prevalence of concussion, at least in part because injuries that they have sustained were not medically identified and documented. If not medically identified, they do not have access to recommended medical management advice, treatment, and rehabilitation^39^ and there might be some who then experience persistent symptoms that are not identified and addressed with evidence-informed treatment. Having multiple variables independently associated with concussion history suggests that future researchers could use different methods to examine the intersectionality of demographic variables and social determinants, with the translational goal of multilevel interventions to improve access to health care for underserved children and adolescents and to, concomitantly, reduce health care disparities.

## Data Acknowledgment and Availability

The dataset used in this study is freely and publically availabe. The citation for the dataset is as follows: Child and Adolescent Health Measurement Initiative (CAHMI) [2021]. 2021 National Survey of Children’s Health, SPSS Indicator dataset. Data Resource Center for Child and Adolescent Health supported by Cooperative Agreement from the U.S. Department of Health and Human Services, Health Resources and Services Administration (HRSA), and Maternal and Child Health Bureau (MCHB). Retrieved September 17, 2023, from www.childhealthdata.org.

## Abbreviations Used

CI: confidence interval
GED: General Educational Development
NSCH: National Survey of Children's Health
OR: odds ratio
SDoH: social determinants of health
SD: standard deviation

## References

[1] Braveman PA, Egerter SA, Mockenhaupt RE. Broadening the focus: The need to address the social determinants of health. Am J Prev Med 2011;40(1 (Suppl 1)):S4–S18; doi: 10.1016/j.amepre.2010.10.002 21146778

[2] Marmot M, Friel S, Bell R, et al. Commission on Social Determinants of Health. Closing the gap in a generation: Health equity through action on the social determinants of health. Lancet 2008;372(9650):1661–1669; doi: 10.1016/S0140-6736(08)61690-6 18994664

[3] Cook NE, Kissinger-Knox A, Iverson IA, et al. Social determinants of health and health equity in the diagnosis and management of pediatric mild traumatic brain injury: A content analysis of research underlying clinical guidelines. J Neurotrauma 2023;40(19–20):1977–1989; doi: 10.1089/neu.2023.0021 37071186 PMC10541940

[4] Tarazi C, Skeer M, Fiscella K, et al. Everything is connected: Social determinants of pediatric health and disease. Pediatr Res 2016;79(1–2):125–126; doi: 10.1038/pr.2015.220 26841091

[5] Viner RM, Ozer EM, Denny S, et al. Adolescence and the social determinants of health. Lancet 2012;379(9826):1641–1652; doi: 10.1016/S0140-6736(12)60149-4 22538179

[6] Braveman P, Egerter S, Williams DR. The social determinants of health: Coming of age. Annu Rev Public Health 2011;32:381–398; doi: 10.1146/annurev-publhealth-031210-101218 21091195

[7] Gottlieb L, Fichtenberg C, Alderwick H, et al. Social determinants of health: What’s a healthcare system to do? J Healthc Manag 2019;64(4):243–257; doi: 10.1097/JHM-D-18-00160 31274816

[8] Bailey ZD, Feldman JM, Bassett MT. How structural racism works - Racist policies as a root cause of U.S. racial health inequities. N Engl J Med 2021;384(8):768–773; doi: 10.1056/NEJMms2025396 33326717 PMC11393777

[9] Kim S, Connaughton DP, Spengler JO. Youth sport parents’ familiarity and perceptions of concussion legislation. J Concussion 2022;6:1–8; doi: 10.1177/20597002221075007

[10] Wallace J, Covassin T, Moran R. Racial disparities in concussion knowledge and symptom recognition in American adolescent athletes. J Racial Ethn Health Disparities 2018;5(1):221–228; doi: 10.1007/s40615-017-0361-1 28389906

[11] Wallace J, Affagato R, Brooke M, et al. Racial disparities in parent knowledge of concussion and recognition of signs and symptoms. J Safety Res 2020;75:166–172; doi: 10.1016/j.jsr.2020.09.007 33334474

[12] Wallace J, Beidler E, Register-Mihalik JK, et al. Examining concussion nondisclosure in collegiate athletes using a health disparities framework and consideration of social determinants of health. J Athl Train 2022;57(1):16–24; doi: 10.4085/1062-6050-0054.21 35040984 PMC8775278

[13] Charleston L, Posas J. Categorizing sports-related concussion disparities by key domains of social determinants of health. Curr Pain Headache Rep 2024;28(3):125–132; doi: 10.1007/s11916-023-01187-2 38227210

[14] Copley M, Jimenez N, Kroshus E, et al. Disparities in use of subspecialty concussion care based on ethnicity. J Racial Ethn Health Disparities 2020;7(3):571–576; doi: 10.1007/s40615-019-00686-6 31898059

[15] Lyons TW, Miller KA, Miller AF, et al. Racial and ethnic differences in emergency department utilization and diagnosis for sports-related head injuries. Front Neurol 2019;10:690; doi: 10.3389/fneur.2019.00690 31312172 PMC6614199

[16] Wallace J, Mannix RC. Racial disparities in diagnosis of concussion and minor head trauma and mechanism of injury in pediatric patients visiting the emergency department. J Pediatr 2021;233:249–254.e1; doi: 10.1016/j.jpeds.2021.01.057 33524386

[17] Nielsen-Bohlman L, Panzer AM, Kindig DA. Health Literacy: A Prescription to End Confusion. Institute of Medicine (US) Committee on Health Literacy: Washington, DC, US; 2004. 25009856

[18] Health Literacy in Healthy People 2030. Available from: https://health.gov/healthypeople/priority-areas/health-literacy-healthy-people-2030 [Last accessed: February 22, 2024].

[19] Lazar M, Davenport L. Barriers to health care access for low income families: A review of literature. J Community Health Nurs 2018;35(1):28–37; doi: 10.1080/07370016.2018.1404832 29323941

[20] Toy J, Gregory A, Rehmus W. Barriers to healthcare access in pediatric dermatology: A systematic review. Pediatr Dermatol 2021;38(Suppl 2):13–19; doi: 10.1111/pde.14748 34338358

[21] Fischer A, Conigliaro J, Allicock S, et al. Examination of social determinants of health among patients with limited English proficiency. BMC Res Notes 2021;14(1):299; doi: 10.1186/s13104-021-05720-7 34353369 PMC8340469

[22] Eneriz-Wiemer M, Sanders LM, Barr DA, et al. Parental limited English proficiency and health outcomes for children with special health care needs: A systematic review. Acad Pediatr 2014;14(2):128–136; doi: 10.1016/j.acap.2013.10.003 24602575

[23] Lawrence EM, Rogers RG, Hummer RA. Maternal educational attainment and child health in the United States. Am J Health Promot 2020;34(3):303–306; doi: 10.1177/0890117119890799 31833396 PMC7033002

[24] Assari S, Caldwell CH, Bazargan M. Association between parental educational attainment and youth outcomes and role of race/ethnicity. JAMA Netw Open 2019;2(11):e1916018; doi: 10.1001/jamanetworkopen.2019.16018 31755951 PMC6902825

[25] Child and Adolescent Health Measurement Initiative (CAHMI). 2021 National Survey of Children’s Health. SPSS Indicator Dataset 2021.

[26] Cheng J, Ammerman B, Santiago K, et al. Sex-based differences in the incidence of sports-related concussion: Systematic review and meta-analysis. Sports Health 2019;11(6):486–491; doi: 10.1177/1941738119877186 31567052 PMC6822209

[27] Merritt VC, Padgett CR, Jak AJ. A systematic review of sex differences in concussion outcome: What do we know? Clin Neuropsychol 2019;33(6):1016–1043; doi: 10.1080/13854046.2018.1508616 30618335

[28] Saadi A, Himmelstein DU, Woolhandler S, et al. Racial disparities in neurologic health care access and utilization in the United States. Neurology 2017;88(24):2268–2275; doi: 10.1212/WNL.0000000000004025 28515272 PMC5567325

[29] Pate J, Cummins I, Mooney J, et al. Socioeconomic and demographic considerations of pediatric concussion recovery. J Clin Neurosci 2022;100:94–99; doi: 10.1016/j.jocn.2022.04.008 35430429

[30] Kuhn AW, Grusky AZ, Cash CR, et al. Disparities and inequities in youth sports. Curr Sports Med Rep 2021;20(9):494–498; doi: 10.1249/JSR.0000000000000881 34524194

[31] Bopp T, Turick RM, Vadeboncoeur JD, et al. Are you welcomed? A racial and ethnic comparison of perceived welcomeness in sport participation. Int J Exerc Sci 2017;10(6):833–844.

[32] Barter EW, Rivera MJ, Post EG, et al. Differences in access to athletic trainers in public secondary schools based on socioeconomic status. J Athl Train 2023;58(2):91–96; doi: 10.4085/1062-6050-0240.21 34623428 PMC10072086

[33] Ramirez N, Shi K, Yabroff KR, et al. Access to care among adults with limited English proficiency. J Gen Intern Med 2023;38(3):592–599; doi: 10.1007/s11606-022-07690-3 35882706 PMC9971409

[34] Himmelstein J, Cai C, Himmelstein DU, et al. Specialty care utilization among adults with limited English proficiency. J Gen Intern Med 2022;37(16):4130–4136; doi: 10.1007/s11606-022-07477-6 35349026 PMC9708984

[35] Lin AC, Salzman GA, Bachman SL, et al. Assessment of parental knowledge and attitudes toward pediatric sports-related concussions. Sports Health 2015;7(2):124–129; doi: 10.1177/1941738115571570 25984257 PMC4332649

[36] Iverson GL, Gaudet CE, Karr JE. Examining suicidality in adolescents who have sustained concussions. J Neurotrauma 2023;40(7–8):730–741; doi: 10.1089/neu.2022.0233 36006376

[37] Iverson GL, Wojtowicz M, Brooks BL, et al. High school athletes with ADHD and learning difficulties have a greater lifetime concussion history. J Atten Disord 2020;24(8):1095–1101; doi: 10.1177/1087054716657410 27431932

[38] Flores G, Abreu M, Tomany-Korman SC. Limited english proficiency, primary language at home, and disparities in children’s health care: How language barriers are measured matters. Public Health Rep 2005;120(4):418–430; doi: 10.1177%2F003335490512000409 16025722 10.1177/003335490512000409 PMC1497749

[39] Patricios JS, Schneider KJ, Dvorak J, et al. Consensus statement on concussion in sport: The 6th international conference on concussion in sport–Amsterdam, October 2022. Br J Sports Med 2023;57(11):695–711; doi: 10.1136/bjsports-2023-106898 37316210

